# A Population Health Surveillance Theory

**DOI:** 10.4178/epih/e2012007

**Published:** 2012-11-30

**Authors:** Farouk El Allaki, Michel Bigras-Poulin, Pascal Michel, André Ravel

**Affiliations:** 1 Groupe de Recherche en Épidémiologie des Zoonoses et Santé Publique (GREZOSP), Université de Montréal, Quebec, Canada.; 2 Laboratory for Foodborne Zoonoses, Public Health Agency of Canada, Quebec, Canada.

**Keywords:** Public health, Animal health, Surveillance concept, Population, Surveillance theory

## Abstract

**OBJECTIVES:**

Despite its extensive use, the term "Surveillance" often takes on various meanings in the scientific literature pertinent to public health and animal health. A critical appraisal of this literature also reveals ambiguities relating to the scope and necessary structural components underpinning the surveillance process. The authors hypothesized that these inconsistencies translate to real or perceived deficiencies in the conceptual framework of population health surveillance. This paper presents a population health surveillance theory framed upon an explicit conceptual system relative to health surveillance performed in human and animal populations.

**METHODS:**

The population health surveillance theory reflects the authors' system of thinking and was based on a creative process.

**RESULTS:**

Population health surveillance includes two broad components: one relating to the human organization (which includes expertise and the administrative program), and one relating to the system *per se* (which includes elements of design and method) and which can be viewed as a process. The population health surveillance process is made of five sequential interrelated steps: 1) a trigger or need, 2) problem formulation, 3) surveillance planning, 4) surveillance implementation, and 5) information communication and audit.

**CONCLUSIONS:**

The population health surveillance theory provides a systematic way of understanding, organizing and evaluating the population health surveillance process.

## INTRODUCTION

Population health surveillance (PHS) represents one of the core functions of public health and animal health and is crucial in the prevention and control of various priority health conditions and diseases. The general objective of PHS is to achieve a timely and continuous assessment of a given population status with respect to a health problem [1,2]. In short, PHS helps fulfill a need for timely and pertinent information.

In this paper, we define the word 'theory' as being a set of interconnected statements, concepts, or propositions linked by a communality of purpose or topic leading to empirical verification [3]. A theory is an attempt to understand, explain and interpret a reality of a phenomenon or a given subject matter as one sees it [3,4]. By definition, a theory is testable and should lead to predictions [3]. The expression 'a population health surveillance theory' refers to a theory integrating all concepts essential to the surveillance process and determines the rules connecting these concepts, it should provide a systematic way of understanding, explaining and organizing surveillance and serve as a global base line. The population health surveillance theory (PHST) should also act as an organizing frame leading to the development of a functional organization (*i.e.*, a PHS program). The PHST makes a prediction on whether or not a given surveillance program is acceptable or not, based on the exhaustivity of the concepts and the coherence of the concept relationships. According to the authors, the term 'population health surveillance' includes the surveillance of population health indicators, diseases, infections, pathogens, risk factors and any factor or determinant that may provide an indication on the health status of a population.

The recent threat of a global influenza pandemic, the incidence of severe acute respiratory syndrome (SARS) cases in many countries, as well as the emergence and re-emergence of many more infectious diseases have demonstrated the prime importance of having effective surveillance programs at the regional, national and international levels [5]. In the new International Health Regulations (IHR), adopted in May 2005 by the 58th World Health Assembly, provision was made for a legal framework designed to prevent, protect, control and provide a public health response against diseases and health events that may constitute a public health emergency of international concern. The new IHR has established a set of rules and procedures to support the existing global outbreak alert and response system, to require countries to improve international surveillance and notification mechanisms for public health events, and to strengthen their national surveillance and intervention capacities. According to the IHR 2005, each World Health Organization (WHO) member state and each country that has agreed to be bound by these regulations shall develop, strengthen and maintain the capacity to detect, assess, notify and report health events in accordance with specific core capacity requirements [5-7].

From an animal health and trade perspective, the World Trade Organization agreement on the application of Sanitary and Phytosanitary Measures (SPS agreement) sets out the basic rules for food safety and animal and plant health standards [8]. The SPS agreement is very clear regarding each country's right to adopt their own sanitary measures to protect the health of their people, animals and plants [8,9]. These measures must be based on international standards or on a scientific assessment of risk [8,9]. As a consequence, the SPS agreement has increased the need for countries to provide science-based evidence to support their claims regarding population health status. This has wide implications as without an effective and reliable surveillance program in operation, a country attempting to export goods would be unable to provide valid scientific and technical information on its health status to an importing country, increasing the risk of losing trade opportunities [8,10].

There are two important knowledge elements underpinning a PHS process: a methodological know-how set of practices and a set of concepts and principles framing the design characteristics of the surveillance program. There are a wide range of methods that are being proposed and applied in the context of PHS and despite the variety of methods and practices proposed, most PHS programs documented seem to rely on an ill-defined conceptual base [1,11-17]. One important concept often left ambiguous relates to delineating the PHS process from the disease control process. In the literature, three different views on this issue have been published: first, the disease control process is different from the surveillance process [18]; second, the disease control process and therefore the intervention strategies are components of the surveillance process [19-21]; and third, the surveillance process is part of any disease control process [1,22,23].

Until 1950, the concept of 'Surveillance' was restricted to the observation of people with serious communicable diseases with the objective of detecting symptoms so that prompt isolation could be instituted [17,24]. In 1963, Alexander Langmuir broadened the use of the term 'Surveillance' by applying it to disease rather than individuals [17,24]. Langmuir limited the scope of surveillance to collection, analysis and dissemination of data in public health practice [17,25-27], thus according to Langmuir's definition, surveillance does not encompass direct responsibility for disease control activities [17,26,27]. In 1966, surveillance was defined by the WHO to include "the epidemiological study of disease as a dynamic process" and a variety of disease control activities [27,28]. This included eight core components for an effective surveillance program, namely: detection; registration; confirmation; reporting; data analysis and interpretation; epidemic preparedness; response and control; and feedback [21]. These components were reviewed by the WHO in 2001 but the disease control activities were still kept within the scope of public health surveillance programs [29]. Other public health researchers proposed activities similar to the ones proposed by the WHO to scope the PHS, but excluded components relating to epidemic preparedness, and the response and disease control activities from their definition [18,30-33].

For animal health specialists, PHS programs only require three components: first, a defined disease monitoring program; second, a predefined disease intervention strategy; and third, a defined threshold of disease frequency [1,19]. In this case, 'monitoring' describes the ongoing efforts directed at assessing the health and disease status of a given population, which necessitates a program for collecting, processing and summarizing data, and disseminating information to appropriate organization as well as individuals [1,11,19].

One of the main consequences of this inconsistent scoping of surveillance and misunderstanding of the driving concepts is the difficulty in establishing rigorous evaluations of surveillance programs. Although we can find a wealth of publications describing the concepts, applications and methods that can be used in the context of PHS, these elements have never been assembled to constitute the base of a PHST [1,11-17,32].

The main objective of this paper is to propose a theory of population health surveillance which would be based on an explicit system of values about health surveillance in a given population.

## MATERIALS AND METHODS

The population health surveillance theory reflects the authors' system of thinking and was based on a creative process.

The authors used the definitions of Carol Grbich for the following concepts: constructivism, positivism and intersubjectivity [34].

## RESULTS

### The broad notion of surveillance

The word 'Surveillance' was originally derived for the French verb 'Surveiller' which literally means 'to watch over'. In this context, "Surveillance" refers broadly to the activity of watching over a person, a population, an object or a phenomenon. More specifically, surveillance is a continuous and dynamic process of close observation of a specific target. Hence, in addition to the notion of 'observation', surveillance should implicitly include the following elements: ongoing data collection, analysis, interpretation, knowledge production, and timely information dissemination (i.e. information sharing) on the state of a pre-defined target to an appropriate audience (e.g. stakeholders, decision-makers), it is therefore essentially a repetitive process aimed at producing targeted information.

### Population health surveillance

PHS refers to the health surveillance of a given population as measured by health or disease indicators while 'individual health surveillance' refers to the description of the health or disease status of a person or an animal. Unlike individual health surveillance, PHS involves a specific population structure (e.g. subpopulations, population at risk) often varying in time and space and can be further seen as a collective activity in consideration of the organized human effort needed for its functioning. This relates to the concept of "organization" in the primary sense of the term, such as a group of people that work together, setting objectives and then judiciously using financial and material resources to achieve them. In short, PHS is a specialized type of organization which performs surveillance on populations. The following observations concerning PHS can also be made:

PHS is a type of organization which enables the production of epidemiological intelligence. As such, one of the central tasks should be the ability to detect and prioritize which hazards should be investigated (problem definition).

PHS also goes beyond simply observing a given population. PHS often involves a specific target (e.g. specific disease and population), specific objective(s) and continuous (repetitive) processes.

**Figure 1:**
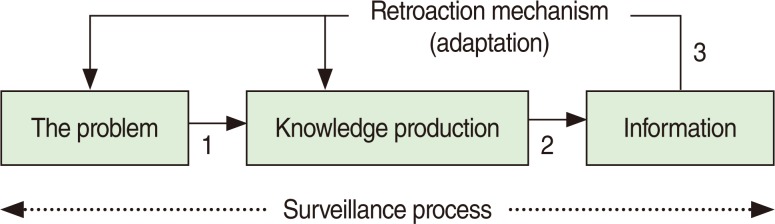
Retroaction aspect of the surveillance process.

PHS often features retroactions on the surveillance process and thus should be considered as a mathematical system (Figure 1). The PHS involves a retroaction (feedback) process where input and output are seen as needs and information respectively. The produced information is then assessed and adjustments (i.e. retroactions) are made, when necessary, to improve a set of attributes describing the surveillance process. Through these retroactions, the surveillance process adapts to the needs of the stakeholders' objectives.

In a modern conceptualization of surveillance, PHS differs from population health monitoring in that the latter involves no or only a crude level of analysis of the collected data which is often directed at detecting changes in disease level or risk factors in a given population. The levels of data analysis and data representation are usually rudimentary in any monitoring process compared to 'Surveillance'. It could be, for example, a simple count of all notified cases, or a repetitive calculation of disease prevalence in a specific region. The Surveillance process generates more, or a richer, knowledge than a monitoring process as it includes the contextual interpretation of this data (to be used for decision). The surveillance process usually involves advanced statistical analysis to generate knowledge. Surveillance is based on an epidemiological intelligence approach, meaning that collected data is processed intelligently to produce knowledge. The use of the word 'Surveillance' or 'Monitoring' will then depend on the complexity of the problem, and the level of information and knowledge to generate. In this context, surveillance includes continuous data collection, the use of intelligent and advanced data analysis techniques, the production of knowledge, and the communication of the information to decision makers in a timely manner. Surveillance implies the use of a well structured communication system, in which monitoring programs could be either absent or rudimentary.

### Population health surveillance process components

**Figure 2:**
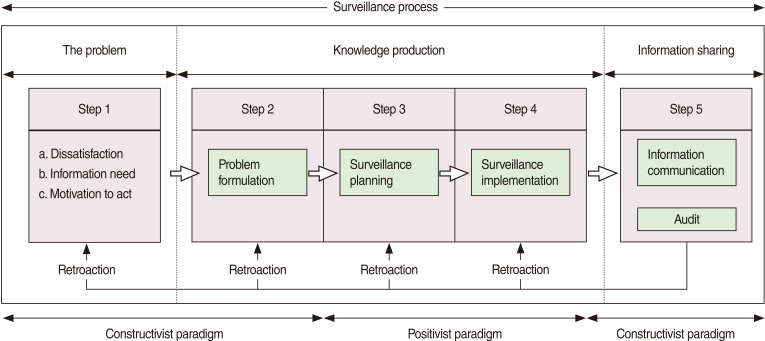
Population health surveillance process components.

The PHS process is composed of five interrelated steps (Figure 2).

#### The problem

The initiation of a surveillance process usually requires the presence of three essential elements: i) a dissatisfaction, ii) a need for knowledge and/or time dependent information, and iii) some level of motivation to eliminate the dissatisfaction and meet the information need about the population health status. There is a need for surveillance as long as these elements persist.

Dissatisfaction is the feeling of psychological discomfort that emerges when a person compares his/her perception of two situations - one representing the actual state of things vs. the desired state of things. Dissatisfaction then is subjective and is part of the affective dimension of a problem. When dissatisfaction is shared by a sufficiently important portion of the decision makers and the stakeholders then it constitutes the starting point for a surveillance initiative. In most instances, the lack of useful information is a source of dissatisfaction and correction of that state is sought.

The coexistence of these three prerequisite elements (i.e. dissatisfaction, need for information, motivation) is the foundation on which the problem is perceived and first scoped. This step most often involves various groups of participants (content experts, stakeholders, decision-makers) and is based on the concept of intersubjectivity of the constructivism paradigm, which is a state of overlap of individual understandings that overemphasizes agreement and de-emphasizes disagreement among the participants. At this point, it is necessary to build an explicit formulation of the problem that can be discussed and enriched until a consensus is reached among the participants.

#### Problem formulation

**Figure 3:**
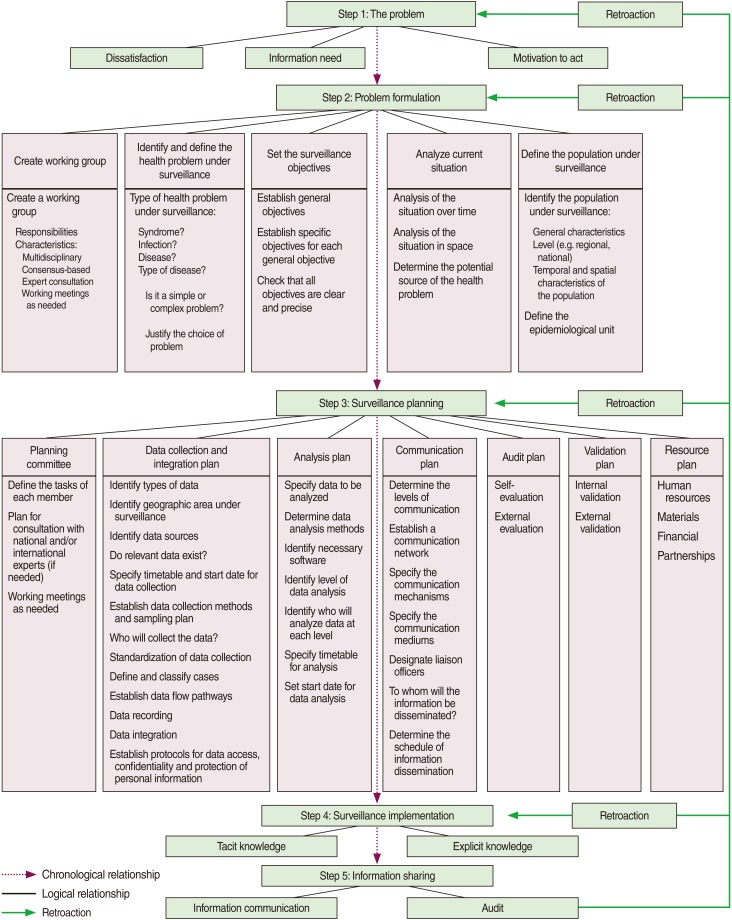
Model detailing the essential components of the population health surveillance process steps.

Problem formulation requires the formal and specific description of the dissatisfaction, which represents the first step in the knowledge production process. Problem formulation is often challenging due to the degree of difficulty relating to the nature of the problem, and the knowledge and experience of the people formulating the problem. The definition of a problem often requires close cooperation and consensus building among various stakeholders working in different disciplines. In the context of PHS, the following elements have to be included during problem formulation: identifying the essential stakeholders and multi-disciplinary expertise, identifying the health outcome to be measured, defining the population under surveillance, analyzing the current situation and available knowledge, and setting the surveillance objectives (Figure 3). The problem formulation process must be clear, rigorous, precise, and based on a constructivist paradigm and intersubjectivity.

#### Surveillance planning

Surveillance planning is defined as an ongoing process that provides a technical and a logistical framework for making decisions concerning the expected results and the strategies available for solving the surveillance problem. Surveillance planning needs to be consistent with the objectives and the frameworks previously defined during the problem formulation step. The final result in surveillance planning is a collective action plan that identifies activities, task division and resource allocation. The following elements must also be considered during the surveillance planning step: data collection and integration (including database issues), data analysis, and information communication. After identifying the main surveillance elements and establishing a plan for each of these activities, it is necessary to link them all together via an administrative and organizational plan (the program). PHS is a collective activity that requires resources: human, financial and material. During the planning stage, the feasibility, the availability of sufficient resources and the compliance with sound methodology should be foreseen. External validation of the proposed plan should be considered. Elaborating an audit process is the final step of the surveillance planning step (Figure 3).

#### Surveillance implementation

Implementing the surveillance program is the final step in the knowledge production process. The objective of this step is to put the surveillance planning into action. Surveillance implementation is an ongoing process of intentional actions aimed at producing two types of knowledge: tacit and explicit. The tacit knowledge is hard to formalize and communicate. However, the explicit knowledge can be encoded, articulated into formal language, shared, stored and communicated through information technologies. The audit of the surveillance program is a crucial element to be taken into account during the surveillance implementation step. The surveillance program evaluation needs to be a recurrent process aimed at comparing the surveillance results with surveillance objectives. As a result of the surveillance evaluation, the problem, the objectives and the surveillance plan may be re-assessed accordingly.

#### Information sharing

The explicit knowledge generated during the knowledge production step is in fact information shared through various communication channels and formats appropriate for the target audience(s). The produced information should be communicated on a regular basis to maintain the motivation and the engagement of all stakeholders and decision-makers. Most often, the frequency by which this information is produced and shared will vary according to the disease epidemiology (e.g. endemic, epidemic/epizootic disease), the socio-economic impact of the health problem, as well as the information needed for disease control activities and international obligations or requests.

## DISCUSSION

The PHST is an explicit theory that helps explain and organize the surveillance process. The first element of the theory describes the context and initial state which triggers the need for a PHS activity and relates primarily to the identification of the hazards and to the feeling of dissatisfaction by stakeholders in face of these hazards [1,35]. The basic questions underpinning this first element are: what hazard(s), to whom, where and when. The second step is directly consequent to this initial state and, as for any scientific process, aims at providing an explicit formulation of the problem which is necessary before the next step can begin [19,36]. The coherence of the surveillance process and related activities will depend directly on the quality of the problem formulation.

The surveillance planning and implementation steps of the surveillance process aims primarily at producing a strategic plan that will frame the production of specific knowledge according to the problem formulation step. Coherence between strategy and objectives is required not only at the onset of surveillance but also during the ongoing execution of the surveillance activities [37]. In this context, it is therefore essential to plan and include an audit and a review of the strategy as retroactive actions. Once the strategy is agreed upon and authorized by the organization mandated to carry out the surveillance, then it must be implemented and executed.

The PHS theory proposes a clear distinction between surveillance and monitoring processes with the latter referring to essentially a data gathering activity with a crude presentation of the data. The theory stipulates that surveillance is based on an epi-intelligence approach and aims to produce knowledge through advanced analysis techniques [12,17]. As an illustration, if we consider a patient in an intensive care unit - the equipment monitors while the medical staff watches over the situation. The monitor refers to the screen on which the patient's data is continuously collected and displayed. PHS is a much more involved activity that will always incorporate some degree of data monitoring. Problem formulation and knowledge production are two essential components of PHS that do not exist to the same extent in monitoring. Monitoring can often be largely automated but it is doubtful that, even in the future, surveillance could ever become fully automated due to the complex nature of data interpretation and feedback actions. In simple terms, monitoring is to be supplemented by intelligent actions in order to be framed as "surveillance". This concept is fundamental to the proposed theory of surveillance.

The PHS activities are directed towards addressing specific population-level problems, and therefore are relevant to a group of individuals (communities, populations) and many stakeholders. This notion implies that an organization is required to plan and conduct the surveillance activity [27]. The social representation and population relevance of this organization must be apparent with respect to the problem formulation (how the issue is framed), the selection of surveillance experts, and the stakeholders engaged and consulted [35]. Thus, the application of an explicit theory relevant to population health surveillance provides an appropriate framework to help in reaching consensus on the problem formulation and on the structure of the organization needed to carry out the surveillance activities.

The ongoing aspect of the surveillance process is usually well accepted as the recognition of health hazards may emerge or shift over time. Given that one important objective of surveillance is to produce knowledge about the hazardous situation and since knowledge, when it is obtained, changes the need for further knowledge, the hazard and the knowledge base need must be continuously reassessed. A rapidly changing situation must be reassessed more often than a slow changing situation, which implies that it is in the nature of the surveillance activity to contain a retroaction mechanism (Figure 1). This makes surveillance an adaptive knowledge production system as defined in Von Bertalanfy's general system theory [38]. The recurrent retroactions between the produced information, the problem definition, and the methods applied in measuring the outcomes are integral parts of the proposed theory.

The proposed theory describes surveillance as a complex organization that handles a process with a non linear dynamic with the objective of producing information that helps to protect the health of the population from hazards, which could be referred to as epidemiological intelligence [17]. According to the proposed theory, surveillance is based on an epidemiological intelligence approach and surveillance programs should be structured as an intelligence organization [17].

One of the major scoping issues in the field of PHS is whether or not to include the disease control process as part of the surveillance. PHST stipulates that the disease control process is not part of the surveillance process and vice versa. The two processes have different objectives and are consequently distinct, which is a vision shared by Yarrow [18]. PHST suggests that surveillance and disease control programs should be kept independent and separate. The proposed theory of surveillance is conceptually linked directly to knowledge theory while disease control is linked to action and decision theory. This major conceptual difference separates ontologically surveillance from disease control. In the case of an audit of surveillance, the main question would be: did the produced knowledge appropriately reduce the original lack of knowledge? The major question on the audit of disease control would be: did the disease control measures reduce the level of the disease in the population? It is quite possible that a good surveillance program will help to conclude that a disease control program is not needed. For example, one can decide to implement a tobacco control program without necessarily developing a cigarette surveillance program or can establish a vaccination program in a high risk population without implementing a surveillance program. When the disease control process is not included in a surveillance process and vice versa then it is easier to evaluate the performances of either the surveillance program or the disease control program or both. However, when these two activities coexist in the same program then it is difficult to know which process needs to be improved in order to eliminate the dissatisfaction. This situation is frequent especially when the disease, health condition or hazards under surveillance have significant impacts on trade or public health. Therefore, it is useful to separate these two processes and propose a theory for each process. They should be distinct but the two processes can be complementary.

The PHST is a construct reflecting the conceptual framework of the authors. The PHST assumes an overall similarity in the conceptual and structural frameworks of animal health and public health programs. This is justified by the desire to embrace the concept of health into one consistent paradigm-The one health concept.

The proposed theory of population health surveillance refers concurrently to a sequential structured process, a human organization and a system. The PHST offers a coherent thought process which explains and justifies the main components of population health surveillance and should help with the development and documentation of coherent population health surveillance programs. The proposed theory could be used as the basis for a conceptual evaluation tool of public health and animal health surveillance programs in a complement to existing methodological evaluation.

### Positivism

Refers to a school of philosophy that affirms that reality lies only in things which can be seen with the eye [34]. The positivism paradigm views truth as absolute and values the original and unique aspects of scientific research [34].

### Constructivism

In this paradigm, reality is viewed as socially and societally embedded in the mind [34]. Reality is changing over time and knowledge is constructed jointly in interaction by the researcher and the researched through consensus [34]. Multiple realities are thus possible [34].

### Intersubjectivity

Refers to a reconstruction of views through interaction with others using oral and written communications [34].
